# ERK expression and its correlation with STAT1 in esophageal squamous cell carcinoma

**DOI:** 10.18632/oncotarget.16902

**Published:** 2017-04-06

**Authors:** Hu Wang, Ying Zhang, Hailong Yun, Shubiao Chen, Yelong Chen, Zhaoyong Liu

**Affiliations:** ^1^ Department of Orthopaedics, First Affiliated Hospital of Shantou University Medical College, Shantou, Guangdong, China; ^2^ Department of Pathology, Shantou University Medical College, Shantou, Guangdong Province, China

**Keywords:** ESCC, ERK, p-ERK STAT1, immunohistochemistry

## Abstract

**Background:**

Esophageal squamous cell carcinoma is one of leading causes of cancer-related deaths in Chaoshan region a high-risk region for esophageal cancer. Extracellular regulated protein kinases (ERK) usually play an important role in cell proliferation and differentiation. However, accumulating evidence has shown that the ERK was aberrantly expressed in cancers and correlated with STAT1 depression.

**Results:**

The activated ERK downregulates STAT1 expression in ESCC cell lines and U0126 increases expression of STAT1. Our immunohistochemistry result also confirms that the expression of ERK inversely correlated with that of STAT1 in ESCC tumors. In addition, a significantly higher expression of ERK/p-ERK was found in ESCC tissues in comparison with case-matched normal esophageal tissues (*p* < 0.05). Moreover, the immunohistochemical analysis demonstrated that ERK expression was paralleled with the differentiation and clinical stage. In 74 patients with follow-up data, those with ERK^low^ tumors survived significantly longer than those with ERK^high^ tumors (*p* = 0.04); patients with ERK^low^/STAT1^high^ tumors had the longest survival (*p* = 0.001).

**Materials and Methods:**

To investigate whether ERK can mediated STAT1 expression in ESCC, we used the MEK plasmid and U0126, a MEK inhibitor, to treat the cell. To further confirm our *in-vitro* study, we detected the ERK, p-ERK and STAT1 expression in 131 ESCC cases and 22 case-matched normal esophageal tissues adjacent to the tumors specimens.

**Conclusions:**

These findings provide pathological evidence that ERK/p-ERK is negatively correlated with STAT1 in ESCC. Our data suggests that inhibition of ERK and/or restoration of STAT1 expression maybe useful therapeutic strategies for ESCC.

## INTRODUCTION

The mitogen-activated protein kinase (MAPK)- extracellular regulated protein kinases (ERK) signaling pathway is a major regulator in a wide range of cellular processes such as cell proliferation, differentiation, survival and motility [1]. ERK activation has been implicated in the pathogenesis and progression of various cancer types, such as cancers of prostate, kidney and colon [2]. ERK represents an attractive anti-cancer therapeutic target for the development of anticancer drugs. However, studies related to the ERK signaling pathway in ESCC are limited.

Signal transducers and activators of transcription (STATs) are critical mediators of cytokine signaling. Upon cytokine stimulation, STATs become phosphorylated by specific receptor kinases, and activated STATs subsequently dimerize and translocate into the nucleus where they regulate gene expression [3]. STAT1 is an important mediator of gamma-interferon (IFNγ), which activates STAT1 by promoting its phosphorylation at tyrosine 701 residue (Y701) [4]. STAT1 also can be phosphorylated at serine 727 (S727) in response to different stimuli or activators, including ERK, IFNγ, lipopolysaccharide and ultraviolet irradiation [5–7]. STAT1 is frequently down-regulated in various types of human cancer, such as breast cancer, head and neck cancer, multiple myeloma and leukemia [8, 9], and these findings are in line with the postulated role of STAT1 as a tumor suppressor.

Esophageal squamous cell carcinoma (ESCC) is the fourth leading cause of cancer deaths and the fifth most common diagnosed cancer in China [10]. ESCC is particularly prevalent in the Chaoshan area where the age-standardized incidence is approximately 7-fold that of the world population [11]. By studying ESCC cell lines and tumor samples collected from the Chaoshan areas, we previously published that STAT1 is a tumor suppressor of ESCC and this protein is frequently down-regulated in ESCC; importantly, the STAT1^low^ phenotype significantly correlates with a worse clinical outcome [12]. In mouse embryonic fibroblasts, it was found that ERK mediates STAT1 phosphorylation at S727 and increases proteasomal degradation of STAT1 [13]. Nonetheless, the expression of ERK/p-ERK in ESCC and its correlation with STAT1 have not been previously explored. With this background, the main purpose of this study is to find out the correlation of STAT1 and ERK/p-ERK in ESCC.

Here, we identified that ERK is negatively correlated with STAT1. The importance of the ERK/STAT1 axis in ESCC is highlighted by the observation that the ERK^high^/STAT1^low^ phenotype significantly correlates with a worse clinical outcome in ESCC patients.

## RESULTS

### ERK mediated STAT1 expression in ESCC cells

**Figure 1:**
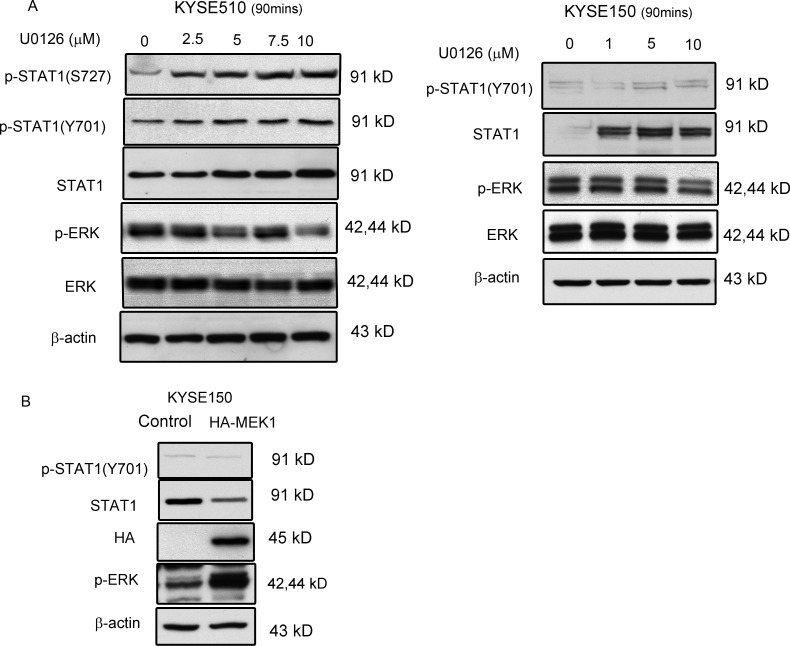
ERK mediated STAT1 expression in ESCC (**A**) KYSE510 and KYSE150 cell lines were treated with increasing doses of U0126 for 90 min. Western blot analysis of p-STAT1, STAT1, p-ERK and ERK in total cell lysates are shown. (**B**) KYSE150 cells were transfected with a constitutive-active MEK expression (HA-ca-MEK) plasmid, and endogenous protein levels of p-STAT1 and STAT1 in the lysates were determined by immunoblotting. Similar results were observed in three independent experiments.

In mouse embryonic fibroblasts, it has been described that ERK mediates phosphorylation of STAT1 at the serine 727 residue and increases proteasomal degradation of STAT1 [13]. Thus, we asked if the expression and activation of STAT1 in ESCC is also regulated by the ERK signaling pathway. In keeping with this hypothesis, treatment of KYSE510 and KYSE150 with varying concentrations (0–10 μM) of U0126, a MEK/ERK inhibitor, increased the levels of STAT1 and p-STAT1 in a dose-dependent manner (Figure 1A). At the same time, the level of p-ERK decreased while the total ERK protein level was unchanged. Since KYSE150 expresses a relatively low level of p-STAT1 serine 727 at the steady state, we can't detect it by using western blots which subjected to a longer exposure. As shown in Figure 1B, transfection of the HA-tagged, MEK1 plasmid resulted in further reduction of STAT1 and p-STAT1 in KYSE150 cell line.

### ERK/p-ERK expression in ESCC specimens

**Figure 2:**
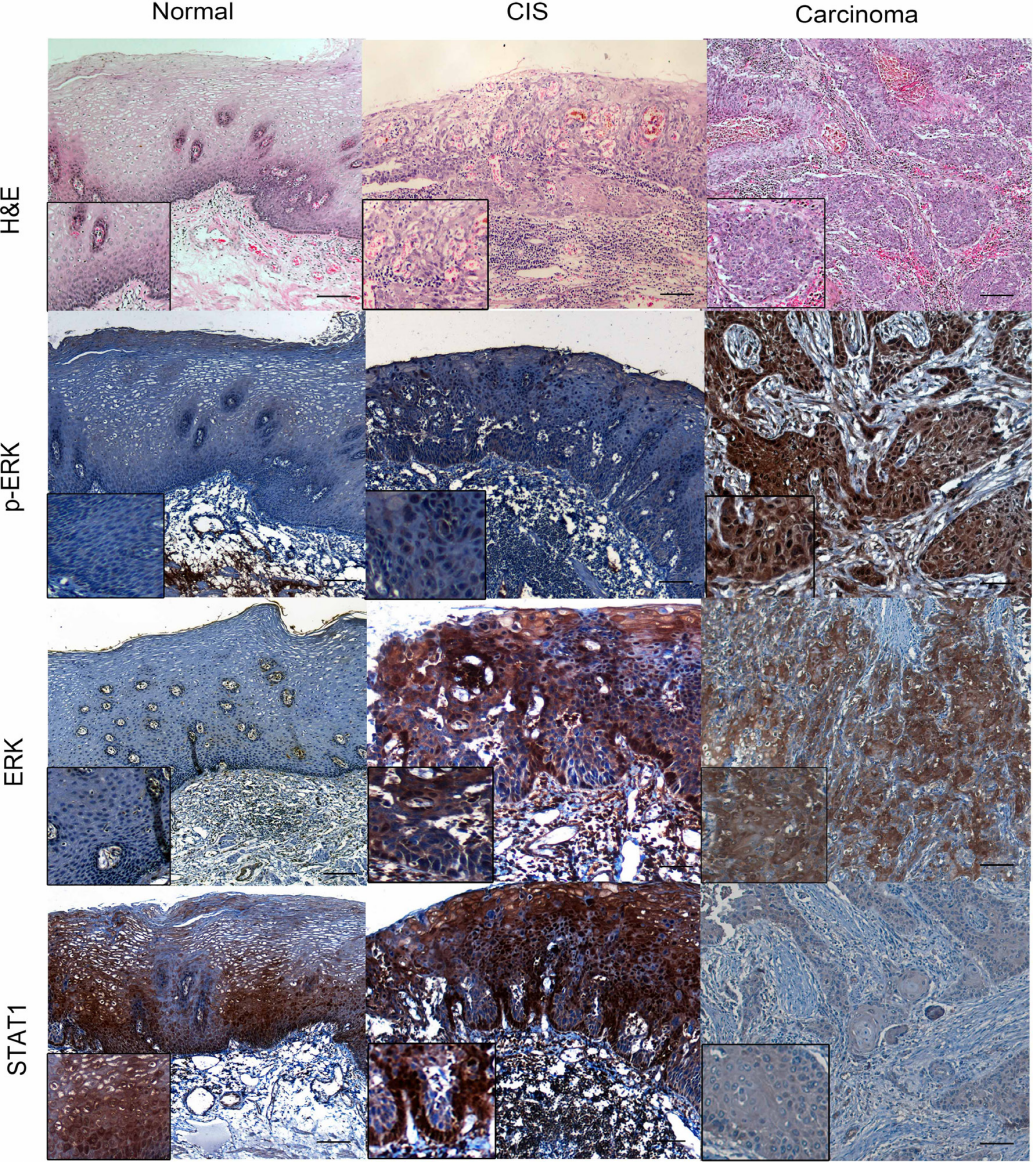
p-ERK, ERK and STAT1 expression in normal epithelial, carcinoma *in situ* (CIS) and ESCC patient samples The esophageal normal epithelial, CIS and carcinoma tissues were stain by Hematoxylin and eosin (H&E). By immunohistochemistry applied to formalin-fixed paraffin-embedded tissues, variable levels of p-ERK, ERK and STAT1 were detected in normal esophageal, CIS and carcinoma examined. (IHC stain, scale bar, 50 μM).

To identify the expression of ERK and p-ERK in ESCC specimens, we performed immunohistochemistry staining in a cohort of patient samples. As shown in Figure 2, the p-ERK expression was mostly found in cell nuclei and ERK expression was found in both nuclei and cytoplasm. The stain intensity was categorized as high and low by immune score. Of these 131 ESCC tissues, there is no detectable ERK in 10 cases and p-ERK in 8 cases. For p-ERK, 66 (50.4%) tumors were assessed high and 65 (49.6%) were low. For ERK, 91 (69.5%) tumors were high and 40 (30.5%) tumors were low. For STAT1, 64 (48.9%) were assessed high and 67 (51.1%) were low, as detected in the previous experiments [12].

### The clinical significance of p-ERK/ERK expression in ESCC

**Figure 3:**
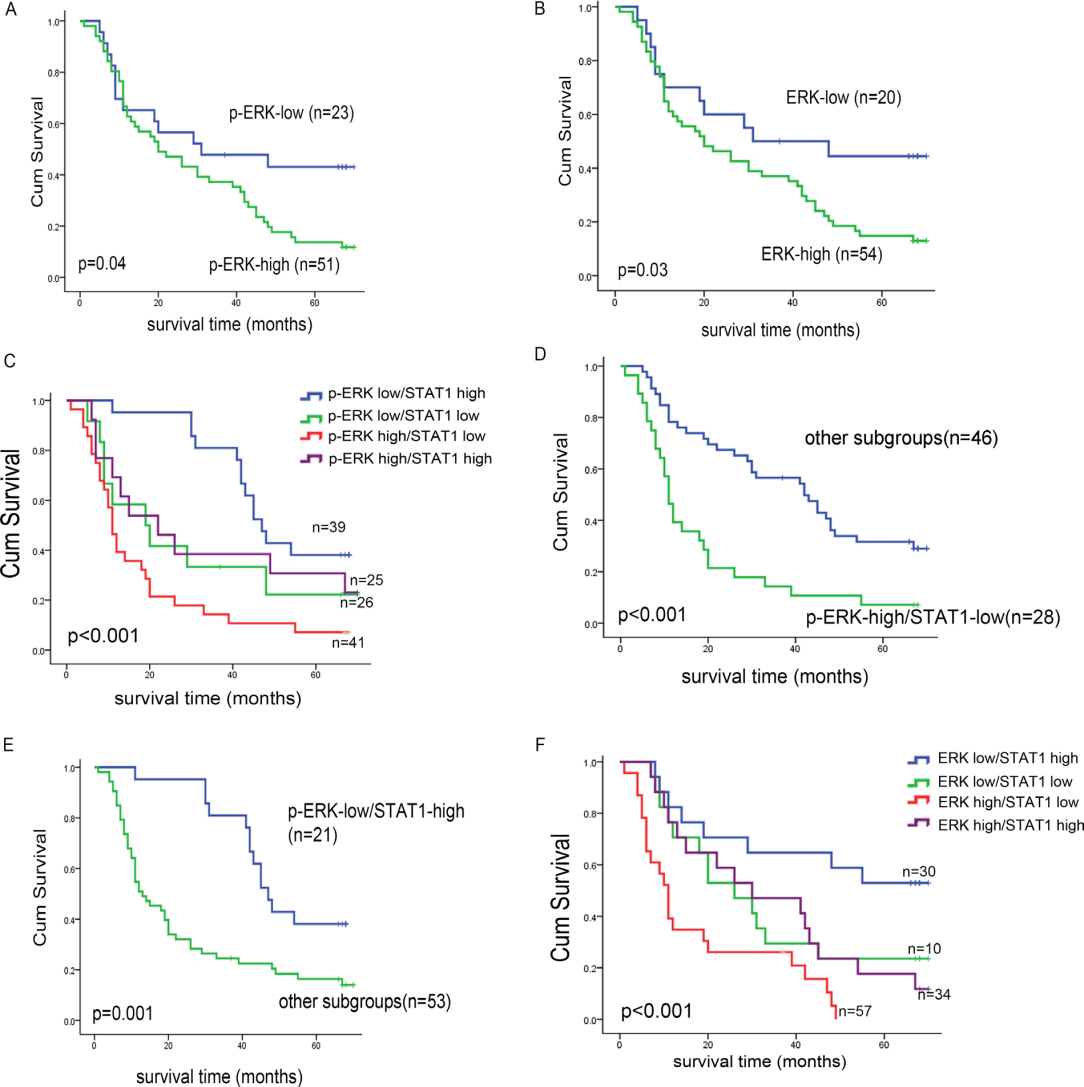
Kaplan-Meier curves of esophageal cancer patients in different p-ERK, ERK and STAT1 sub-groups (**A**) By Kaplan-Meier analysis, the significant correlation between overall survival and the expression level of p-ERK was found, when the two groups were defined as p-ERK^high^ and p-ERK^low^. Similar result was obtained in ERK high/low expression patient groups (**B**). The cohort patients were divided into four sub groups by the STAT1 and p-ERK expression level, we found that patients with p-ERK^low^/STAT1^high^ survived significantly longer than other subgroups (**C**) and those with p-ERK^high^/STAT1^low^ survived significantly shorter than others (**D**). By Kaplan-Meier analysis, significant correlation between overall survivals was found when the cohort patients were divided into four sub groups by the STAT1 and p-ERK/ERK expression level (**E**, **F**).

In our previous study, we found that the expression of STAT1 is prognostically significant [12]. As summarized in Table 1, we correlated p-ERK/ERK expression with various clinical and pathologic parameters and found that both p-ERK and ERK significantly correlated with the clinical stage (*p* = 0.02 and *p* = 0.03, respectively, Chi square) as well as the histologic grade (*p* = 0.04 and *p* = 0.01, respectively, Fisher's exact test). We further performed multivariate analysis to evaluate a number of relevant prognostic factors of ESCC, as shown in Table 2. Poor cell differentiation and high p-ERK staining were found to be independent risk factors for ESCC patients (relative risk = 1.45 and 2.27, respectively). Patient survival is illustrated in Figure 3A and 3B, and we found that patients with p-ERK^low^ tumors survived significantly longer that those with p-ERK^high^ (39.5 months versus 29.0 months, *p* = 0.04). Similar results were obtained when ERK was used instead of p-ERK. Then, we analyzed the survival of these 74 patients categorized into 4 groups based on the differential expression of p-ERK/STAT1, and we found that there is a significant difference (Figure 3C). Specifically, patients with tumors that are p-ERK^low^ and STAT1^high^ expression survived significantly longer than the other 3 groups. Furthermore, patients with tumors that were p-ERK^high^/STAT1^low^ carried the worst prognosis (Figure 3D and 3E). Similar results were obtained when ERK was used instead of p-ERK (Figure 3F).

**Table 1:** Correlations between p-ERK and ERK expression and various clinicopathologic parameters in ESCC

Parameter		Case NO.	low/high expression	
			p-ERK	ERK
Age	≤ 57	66	38/28	20/46
	> 58	65	27/38	20/45
Gender	Male	98	48/50	31/67
	Female	33	17/16	9/24
Tumor site	Upper	13	4/9	3/10
	Middle	104	54/50	33/71
	Lower	14	7/7	4/10
Differentiation	Poor	12	4/8*0.04	2/10*0.012
	Intermediate	75	37/38	20/55
	Well	44	30/14	18/16
Tumor size	> 5 cm	82	40/42	25/57
	< 5 cm	49	25/24	15/34
Depth of invasion	T1–T2	103	55/48	28/75
	T3–T4	28	10/18	12/16
Lymph metastasis	Yes	68	31/37	18/50
	No	63	34/29	22/41
Clinical Stage	1	6	4/2*0.02	5/1*0.031
	2	55	35/20	17/38
	3	64	25/39	17/47
	4	6	1/5	1/5

^*^ *p* < 0.05.

**Table 2:** Univariate and multivariate Cox proportional hazard analyses for cancer-specific survival

	Univariate		Multivariate	
	HR (95% CI)	*P* value	HR (95% CI)	*P* value
**Age, years** (< 57 vs. > 58)	0.94 (0.40–2.23)	0.19		
**Gender** (male vs. female)	0.75 (0.39–1.44)	0.32		
**Tumor site** (middle vs. others)	0.89 (0.25–3.21)	0.50		
**Differentiation** (poor vs. others)	3.82 (1.5–6.9)	0.001	1.45 (0.45–3.43)	0.01*
**Tumor size** (> 5 cm vs. < 5 cm)	1.32 (0.75–2.70)	0.03	0.78 (0.45–1.46)	0.53
**Depth of invasion** (T1–2 vs. T3–4)	0.51 (0.05–5.17)	0.10		
**Lymph node metastasis** (yes vs. no)	1.40 (0.78–3.54)	0.02	0.65 (0.34–1.33)	0.43
**Clinical Stage** (1, 2 vs. 3,4)	0.31 (0.24–1.83)	0.36		
**p–ERK expression** (High vs. low)	4.45 (2.96–6.75)	0.001	2.27 (1.67–4.32)	0.02*
**ERK expression** (High vs. low)	0.95 (0.87–2.54)	0.24		
**STAT1 expression** (low vs. High)	1.43 (0.53–3.43)	0.05	0.88 (0.44–1.21)	0.23

^*^ *p* < 0.05.

### The up-regulation of p-ERK/ERK and down-regulation of STAT1 are implicated in cancer progression of ESCC

**Figure 4:**
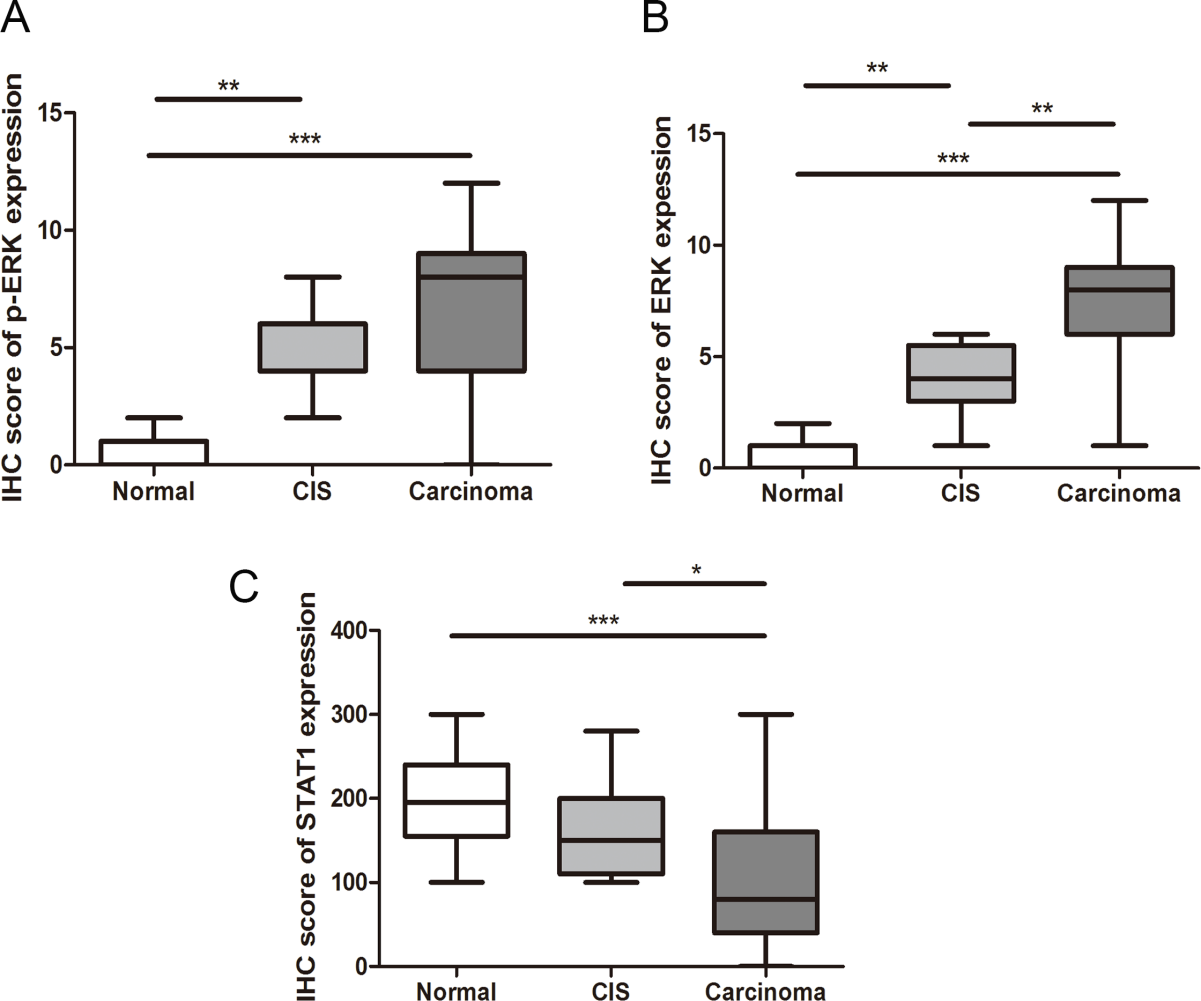
The IHC score of p-ERK (**A**), ERK (**B**) and STAT1 (**C**) in normal tissues, CIS and ESCC are shown. (*p < 0.05, **p < 0.01, ***p < 0.001.

To identify if the up-regulation of p-ERK/ERK and the down-regulation of STAT1 are implicated in ESCC cancer progression, we assessed the expression of these three markers in 22 cases of benign esophageal epithelial tissues adjacent to ESCC and 12 carcinoma *in situ* samples using immunohistochemistry. As illustrated in Figure 4, the expression of p-ERK as well as ERK were significantly lower in benign epithelial tissues (IS: 0.49 ± 0.21), as compared to CIS (IS: 5.47 ± 0.32, *p* < 0.001). The expression of p-ERK in ESCC *(n* = 131) was also higher than that of CIS tissues, although the difference did not reach statistical significance (*p* = 0.10). A similar pattern was found with ERK expression, with gradual decrease from ESCC, CIS to benign esophageal tissues (IS: 7.59 ± 0.26 versus 3.83 ± 0.49 versus 0.52 ± 0.16; *p* < 0.001). Lastly, the expression of STAT1 was significantly higher in benign esophageal tissues (IS: 194.1 ± 11.93) as compared to CIS (IS: 160 ± 15.84) (*p* = 0.04), whereas the STAT1 expression level in CIS was also higher than that of ESCC (IS: 106 ± 7.78), although this difference did not reach statistical significance (*p* = 0.10).

### STAT1 inversely correlates with ERK/p-ERK in ESCC tumors

Results from our previous studies led us to hypothesize that the expression of ERK/p-ERK may inversely correlate with that of STAT1 in ESCC tumors. As shown in Table 3, STAT1 inversely correlated with p-ERK and ERK (*p* = 0.015 and *p* = 0.001, respectively, Fisher's exact test). Analysis of our data also indicated a significant correlation between ERK expression and p-ERK expression (Spearman coefficient, *r* = 0.746; *p* = 0.001).

**Table 3:** The significant correlation between STAT1 and p-ERK/ERK in ESCC

STAT1 expression level	p-ERK expression level			
	High	Low	Total	
**High**	25	39	64	
**Low**	41	26	67	
**Total**	66	65	131	*P* = 0.015*
**STAT1 expression level**	**ERK expression level**			
	**High**	**Low**	**Total**	
**High**	34	30	64	
**Low**	57	10	67	
**Total**	91	40	131	*P* = 0.001*

*p* < 0.05.

## DISCUSSION

STAT1, the first member discovered in the family of STAT proteins, was identified as the key mediator for type I and type II IFNs. Accumulating evidence suggested that STAT1 is a tumor suppressor in various cancer models [3, 14]. STAT1 was found to be frequently down-regulated in neoplastic cells, as compared to their adjacent benign tissues in breast cancer, colorectal cancer and liver cancer [15]. Our group also has recently published that STAT1 plays a tumor suppressor role in ESCC [12], in which we found that 43 of 57 (75.4%) ESCC samples examined showed a down-regulation of STAT1 as compared to the case-matched, benign epithelia adjacent to the tumors. In the same study, we found that patients with STAT1 low tumors had a significantly worse clinical outcome; gene transfection of STAT1 into ESCC cells was found to effectively induce apoptosis, highlighting the biological importance of STAT1 in this tumor.

The ERK pathway is an important, highly conserved family of enzymes associated with cell membrane receptors and regulative targets. An activated ERK cascade controls cell proliferation, differentiation, survival, and apoptosis as well as regulating the cell cycle [16–20]. These cascades also regulate cell meiosis, mitosis, and post mitotic processes in differentiated cells by phosphorylating several molecules, including transcription factors, cytoskeleton elements, apoptosis regulators, translation regulators, and a variety of other signaling-related molecules [21].

The function of ERK in cell proliferation and differentiation has been well documented in various papers. An aberrant ERK cascade activation occurs in a high proportion of human cancers, including human renal cell carcinoma and prostate cancer [22–24]. The inappropriate activation of upstream molecules, such as EGFR, Raf, and Ras, constitute major forces controlling the constitutive activation of the ERK pathway in tumor cells [25–30]. Research identifies this pathway as a promising chemotherapy target. Specifically, the inhibition of the ERK pathway represents an efficient way of blocking proliferation, metastasis, and angiogenesis in cancer cells [31]. Since ERK fulfills an essential role in responding to hepatocyte growth factor (HGF) [32], the obstruction of this pathway downregulates the hypoxia-inducible factor 1a (HIF-1a) [33, 34], a master regulator of angiogenesis, as well as MMP-3/−9/−14 and CD44, important regulators of cell invasion [35]. The expression of ERK in esophageal cancer has been demonstrated in a limited papers. In a cohort of patients from the Kazakh population, researchers have found that the expression of ERK is higher in ESCC samples compared to normal tissue, and this up-regulation significantly correlated with lymph nodes metastasis and histologic grade [36]. In this paper, we found high p-ERK expression was a significant risk factor for ESCC patients, however, the total ERK expression did not show significance. The possible reason is that the phosphorylation at both the threonine 202 and tyrosine 204 residues of ERK1 or threonine 185 and tyrosine 187 residues of ERK2 is required for full enzymatic activation of ERK [37]. The activation of ERK plays a more important role in the malignant of ESCC rather than the total expression of ERK.

Based on previous study with MEF cell lines in which ERK was found to promote STAT1 proteasome degradation in mouse embryonic fibroblasts [13], we tested if ERK expression is negatively correlated with STAT1 expression in ESCC. Our data supports this concept. To our knowledge, this current study represents the first example in which ERK is shown to be negatively correlated with STAT1, a tumor suppressor, in human cancers. While the involvement of ERK mediated the expression and activation of STAT1 has not been previously reported in human cancer models. Based on the results of our study, one may hypothesize that the expression and activation of STAT1 may be closely linked to the expression and activation status of ERK. If this turns out to be the case, one may hypothesize that we can downregulate the ERK activation in order to active STAT1, a apoptosis promoter, to kill the tumor cells.

In conclusion, we have demonstrated that ERK activation in ESCC is negatively correlated with STAT1 expression. In conjunction with our previous publication that STAT1 is a tumor suppressor in ESCC, results from this study strongly supports the concept that ERK suppresses the expression of STAT1 in ESCC. This concept is supported by our clinical observation that the expression of ERK significantly correlates with a worse clinical outcome.

## MATERIALS AND METHODS

### Patient samples

We randomly collected 131 consecutive ESCC tumors at the Shantou Tumor Hospital between 2005 and 2012. All patients underwent potentially curative surgery without preoperative chemotherapy or radiotherapy. In this cohort, 98 were men and 33 were women; the range of age was 36–78 years, with a median of 57 years. Follow-up data was available for 74 patients; most (58, 78.4%) died during the follow-up period (median survival, 31.4 months). Of the 131 ESCC tumors, 22 case-matched normal esophageal tissues adjacent to the tumors were included in the study. Written informed consents were obtained from patients, and the study was reviewed and approved by the institutional ethics committee.

### Immunohistochemistry (IHC)

IHC staining was performed using the Envision Labeled Peroxidase System (Dako, Carpentaria, CA). Consecutive 4 μm thick slices from each sample were deparaffinized in dimethyl benzene, rehydrated through a graded ethanol series and incubated with fresh 3% H_2_O_2_ for 10 minutes to quench endogenous peroxidase activity. After a rinse in phosphate-buffered saline (PBS), antigen retrieval involved microwave heating. Following incubation with 10 mmol/L citrate buffer (pH 6.0) for 20 minutes, primary antibodies for STAT1 (Cell Signaling, 1:75), phospho-p44/42 MARP (ERK1/2,D13.14.4E) (Cell Signaling, 1:200) and p44/42 MARP (ERK1/2, 137F5) (Cell Signaling, 1:500) were incubated at 4°C overnight. After washing, corresponding secondary antibody (DAKO, Carpentaria, CA) was added for incubation at 37°C for 30 minutes before reaction with diaminobenzidine and counterstaining with haematoxylin. Two pathologists who were blinded to the clinical outcome examined IHC staining and consensus between the two pathologists in discrepant cases was reached by performing a review under a double-headed microscope. For the evaluation of STAT1 immunostaining, both the intensity and percentage of immunostained cells were described previously [12]. Scoring for the p-ERK and ERK immunostaining was performed as follows: for the proportion of positivity, each case was categorized as: 0 (< 5% positive cells), 1+ (6–25% positive cells), 2+ (26–50% positive cells), 3+ (51–75% positive cells) or 4+ (> 75% positive cells). Each case was categorized as 0 to 3+ based on the average staining intensity. The final scores were based on the multiplication of the proportion score and intensity score, which ranged from 0 to 12. Tumors were considered ERK^low^ or p-ERK^low^ when they were assigned a score of < 6, whereas tumors were considered ERK^high^ or p-ERK^high^ if they had a final score of ≥ 6.

### Cell lines

Three human ESCC cell lines, KYSE510 and KYSE150, were used in this study. They were maintained in RPMI 1640 supplemented with 10% fetal bovine serum and 1 × antibiotic mixture (Invitrogen, Carlsbad, CA, USA). All cells were cultured at 37°C in a humidified incubator containing 5% CO_2_.

### Plasmid and drug

The pBABE-MEK-1 (HA-MEK1) vector was Purchased from the Addgene. Mitogen-activated protein kinase kinase (MEK1) inhibitor U0126 was purchased from Cell Signaling (Danvers, MA, USA).

### Westernblot and antibodies

The preparation of cell lysates for Western blots was done as follows: cells were washed twice with cold phosphate-buffered saline (PBS, pH = 7.0), and scraped in RIPA lysis buffer (150 mM NaCl, 1% NP-40, 0.5% deoxycholic acid, 0.1% SDS, 50 mM Tris pH 8.0) supplemented with 40.0 μg/mL leupeptin, 1 μM pepstatin, 0.1 mM phenylmethylsulfonyl-fluoride and sodium orthovandate. Cell lysates were incubated on ice for 30 minutes and centrifuged for 15 minutes at 15000 g at 4°C. Proteins in the supernatant were then extracted and quantified using the bicinchoninic acid protein assay (Pierce, Rockford, IL). Subsequently, cell lysates were then loaded with 4× loading dye (Tris-HCl pH 7.4, 1% SDS, glycerol, dithiothreitol, and bromophenol blue), electrophoresed on 8% or 10% SDS-polyacrylamide gels, and transferred onto nitrocellulose membranes (Bio-Rad, Richmond, CA, USA). After the membranes were blocked with 5% milk in Tris buffered saline (TBS) with Tween, they were incubated with primary antibodies. After washings with TBS supplemented with 0.05% Tween-20 for 30 minutes between steps, secondary antibody conjugated with the horseradish peroxidase (Jackson Immunoresearch Laboratories, West Grove, PA, USA) was added to the membrane. The following antibodies were employed: anti-STAT1 (1:1000) and anti-p-STAT1(Tyr-701) (1:1000) and anti-p-STAT1(Ser-701) were purchased from Cell Signaling (Danvers, MA, USA). anti-β-actin (1:1000), anti-phospho-ERK (or p-ERK) (1:1000), anti-ERK (1:1000) and anti-HA, were obtained from Santa Cruz Biotechnology (Santa Cruz, CA, USA). Densitometric analysis was performed using the ImageJ analysis system (Bethesda, WA, USA); the values for the STAT1 bands were normalized to those of the β-actin bands.

### Statistical analysis

Statistical analysis was performed with the SPSS15.0 software. The correlation between ERK/p-ERK and other clinical parameters was evaluated using Chi-square or Student's *t-test*. A value of *p* < 0.05 was considered as statistically significant.
